# Medical Service in the Navy

**Published:** 1843-06-24

**Authors:** 


### THE MEDICAL EXAMINER.

PHILADELPHIA, JUNE 24, 1843.

### MEDICAL SERVICE IN THE NAVY.

According to the Navy Register, (corrected until the first of April, 1843, and recently published by Benjamin Homans, of the Navy Department,) the medical force of the navy consists of 69 surgeons, 18 passed-assistant surgeons, and 48 assistant surgeons, to which number three have been recently added, making one hundred and thirty eight, the aggregate number of physicians engaged in the naval service.

All the surgeons have been long in the navy, and many of them ought to have leave to retire, on the plea of length of service. For example, they stand in the following order. Of the 69 surgeons,

| | | | |
|---|---|---|---|
| 1 | has served | 40 | years and upwards. |
| 10 | have served | 30 | " " |
| 12 | " | 20 | " " |
| 13 | " | 15 | " " |
| 32 | " | 10 | " " |
| 1 | " | 9 | " " |

Of the 18 passed-assistant surgeons,

| | | | |
|---|---|---|---|
| 1 | has served | 14 | years and upwards. |
| 4 | have served | 9 | " " |
| 5 | " | 8 | " " |
| 8 | " | 6 | " " |

Of the 51 assistant surgeons,

| | | | |
|---|---|---|---|
| 4 | have served | 6 | years and upwards. |
| 21 | " | 5 | " " |
| 8 | " | 3 | " " |
| 4 | " | 2 | " " |
| 11 | " | 1 | " " |
| 3 | are recently appointed. | | |

In June, 1842, there were fifteen gentlemen (see Medical Examiner, for June, 1842,) reported as possessing, in a competent degree, the qualifications requisite for discharging the duties of assistant surgeon in the navy, of which number the first three have been appointed since the first day of April, 1843, so that there will be no board of examination for admission into the navy for some time, unless those now waiting for commissions should decline them when offered.

Twenty-five assistant surgeons are now eligible to examination for promotion; and if they were midshipmen with similar claims, we might predict, with certainty, the convening of a board in the course of a few weeks. But as they are "only doctors," and as it is generally believed they may be treated unjustly with impunity, it is uncertain how the department will act; perhaps it will suit its convenience without regard to any other consideration.

We heard a verbal discussion, between a citizen and a lieutenant of the navy, as to the sense of propriety or taste displayed in the letters published by certain Commodores, against the chief of the medical bureau of the navy; the citizen condemned the course pursued by those gentlemen, as injurious to the general interests of the navy and derogatory to themselves, in making a personal attack upon a fellow officer—as they were old officers, their example might influence the younger members of the navy. "Oh! sir," said the Lieutenant, "you must remember that Commodore ——— is a post-captain in the navy, and the man he exposes is nothing but a——Doctor." The citizen expressed an opinion that at any rate, as far as the personal differences were concerned, a doctor was as good and as worthy a man as a captain, and entitled to fully as much justice; and further, that society at large felt as much interest for one as for the other, especially in times of peace. From our intercourse with medical officers of the navy, we have come to the conclusion, they are but too generally regarded by sea officers, the older ones especially, as of an inferior race, and not entitled to be measured by the same measure as themselves. This imperfect view of themselves and their relative position arises from defective education, in which they imbibe the notion, that there is nothing on the face of the land or sea quite equal to the captain of a man-of-war, whether on his own quarter deck or not; consequently those who grow up with such an egregiously wrong notion, and come to be captains, arrogate to themselves what they habitually and reverentially paid, when young, to their commanders. They become incapable of seeing matters of a personal nature, in the same view as others see them—and they are puzzled to comprehend, through what process of misfortune or folly, medical officers of the army have been assigned a rank equivalent to commander in the navy. "It may possibly do in the army," say they, "but in the navy it must not, and, if we can help it, it shall not be." We have been told of a captain who, (although very partial, generally, to the society of the surgeon associated with him on duty,) declared that he would not give him any rank, nor would he give him a sleeping berth on ship board, till all his lieutenants and purser had made their selection of rooms. And we have heard of a lieutenant of the navy, who said that he would not mess at the same table with an assistant surgeon, in the ward-room of a ship of war, unless compelled to do so by an act of Congress, but under such authority he would do so cheerfully; he did not object to the doctor as an associate, but he was totally opposed to the "new fangled notion of making assistant surgeons ward room officers." Some years ago a distinguished captain, who has been long dead, expressed his opinion that a post-captain should always preside at medical boards of examination, to give them dignity!

Notwithstanding the opinion of which the above anecdotes are indicative, we are credibly informed, the moment a captain is assigned to command a ship, he becomes solicitous about having a surgeon ordered, and most frequently claims the privilege of selecting one from the list, and often, too, without consulting the individual he may fancy.

Of the 69 surgeons, 2 are sick, 26 are in vessels on foreign service, 22 are employed in receiving vessels, rendezvous, navy yards and hospitals, &c., and 19 are waiting orders, of whom fourteen, we are told, are infirm and incapable of service.

Of the 18 passed-assistant surgeons, 1 is sick, and 4 on leave, and 13 employed in active service.

Of the 51 assistant surgeons, 1 is sick, and 4 are on leave of absence, having recently returned from foreign duty.

As it cannot be expected, that the same officers should serve constantly out of the United States, there should be a sufficient number to afford them relief, in order to equalize the service, which, heretofore, has not been the case. While some surgeons have been kept more than half the time at sea, a few have performed almost their whole service in the United States, and in some rare instances always on the same spot. This has been mentioned as a grievance that has probably arisen from the number of surgeons being too small for the amount of sea-service required of them—as there is no established routine of sea and shore service equally applicable to all, the sea duty has fallen more heavily upon a certain set.

Duty must be more constantly required of surgeons than of several grades of officers is very evident by comparing them with others on the navy register. For example, we find there are 68 captains, of which number 31 are unemployed, consequently there are 31 waiting to take the places of the 37 who are employed, and of this number but 13 are commanding vessels at sea.

We find there are 96 commanders, of which number 16 are commanding vessels at sea, 26 are on duty in the United States, and 54 are unemployed, or waiting to relieve the 42 who are on service.

The usual distribution of medical officers is as follows:

To ships of the line, one surgeon and three assistants.

To frigates, one surgeon and two assistants.

To sloops of war, one surgeon and one assistant.

To brigs and schooners one passed assistant, or an assistant surgeon.

To navy yards one surgeon and one assistant.

To hospitals, one surgeon and one assistant.

To rendezvous, one surgeon.

According to the navy register, the naval service requires, for active duty at this time, 49 surgeons and 67 assistants, consequently there is not a sufficient number in the navy to afford an adequate relief to those now employed.

We gather from an examination of the navy register that there is a surgeon employed in company with every captain and every commander, the aggregate number of which is 164, so that instead of being 69 surgeons, there ought to be, we should suppose, at least one hundred, and perhaps one hundred and thirty or forty assistants surgeons to make their duties in proportion relatively to the other grades. But this is a question for the decision of the government, and not properly suitable for discussion in our columns.

The amount of duty required from medical officers may be gathered from the fact, that the sick, in our ships afloat, amount, on an average, to five per cent. of the whole number on board. So that, in round numbers, it may be set down, that the number of patients daily requiring attention, is 50 in ships of the line, 25 in frigates, 10 in sloops of war, and 4 in brigs, schooners and store vessels. In vessels where there is more than one medical officer, one medical officer is required to be constantly on board, in readiness to treat any case that may occur.

The cruises are usually for three years, which is a long time to be pent up in a ship, absent from friends and home, constantly engaged in discharging the duties of an arduous profession. And certainly such service is worthy of greater emolument and consideration than are now given to medical men in the navy.

If the medical services were paid for in the navy at fifty cents a visit, bearing in mind that the regulations of the service require the sick to be visited twice daily, they would cost the government as follows:—for a ship of the line, $50 a day; for a frigate, $25; for a sloop, $10, and smaller vessels $4 a day. Of course this could not be the principal of regulating remuneration, although it shows how inadequate the pay of medical officers in the navy is, and is one illustration of the small money value of the profession, which we suspect is rarely considered by those who aspire to its honors.

